# A numerical investigation of intrathecal isobaric drug dispersion within the cervical subarachnoid space

**DOI:** 10.1371/journal.pone.0173680

**Published:** 2017-03-15

**Authors:** Per Thomas Haga, Giulia Pizzichelli, Mikael Mortensen, Miroslav Kuchta, Soroush Heidari Pahlavian, Edoardo Sinibaldi, Bryn A. Martin, Kent-Andre Mardal

**Affiliations:** 1 Center for Biomedical Computing, Simula Research Laboratory, Fornebu, Norway; 2 Istituto Italiano di Tecnologia, Center for Micro-BioRobotics, Pontedera, Italy; 3 Scuola Superiore Sant’Anna, The BioRobotics Institute, Pontedera, Italy; 4 Dept. of Mathematics, University of Oslo, Oslo, Norway; 5 Conquer Chiari Research Center, Dept. of Mech. Engineering, University of Akron, Akron, Ohio, United States of America; 6 Dept. of Biological Engineering, The University of Idaho, Moscow, Idaho, United States of America; Szegedi Tudomanyegyetem, HUNGARY

## Abstract

Intrathecal drug and gene vector delivery is a procedure to release a solute within the cerebrospinal fluid. This procedure is currently used in clinical practice and shows promise for treatment of several central nervous system pathologies. However, intrathecal delivery protocols and systems are not yet optimized. The aim of this study was to investigate the effects of injection parameters on solute distribution within the cervical subarachnoid space using a numerical platform. We developed a numerical model based on a patient-specific three dimensional geometry of the cervical subarachnoid space with idealized dorsal and ventral nerve roots and denticulate ligament anatomy. We considered the drug as massless particles within the flow field and with similar properties as the CSF, and we analyzed the effects of anatomy, catheter position, angle and injection flow rate on solute distribution within the cerebrospinal fluid by performing a series of numerical simulations. Results were compared quantitatively in terms of drug peak concentration, spread, accumulation rate and appearance instant over 15 seconds following the injection. Results indicated that solute distribution within the cervical spine was altered by all parameters investigated within the time range analyzed following the injection. The presence of spinal cord nerve roots and denticulate ligaments increased drug spread by 60% compared to simulations without these anatomical features. Catheter position and angle were both found to alter spread rate up to 86%, and catheter flow rate altered drug peak concentration up to 78%. The presented numerical platform fills a first gap towards the realization of a tool to parametrically assess and optimize intrathecal drug and gene vector delivery protocols and systems. Further investigation is needed to analyze drug spread over a longer clinically relevant time frame.

## Introduction

Intrathecal drug and gene vector delivery (IT) to the central nervous system (CNS) is a procedure involving the release of therapeutic agents into the cerebrospinal fluid (CSF) via an inserted catheter [1]. The CSF is a water-like fluid that resides in the subarachnoid space (SAS) surrounding the brain and spinal cord and is also contained within four fluid filled reservoirs within the brain called ventricles. Total CSF volume in an adult is approximately 150 *ml* [2] with ≃80 *ml* contained within the spinal SAS [3]. CSF in the SAS is bounded on the outside by the arachnoid membrane and dura and on the inside, covering the CNS tissue surface, by the delicate pia mater.

Traditional oral or parenteral drug administration for CNS diseases is limited, mainly due to the shielding effect of the blood-brain barrier to macromolecules. Conversely, thanks to the proximity of the CSF to the brain and spinal cord parenchyma, IT allows many drugs to directly penetrate into the CNS tissue by the leptomeningeal spaces [4–7] thereby requiring a lower drug dosage and resulting in less potential toxic effects [8, 9].

The strong interest in IT stems from the fact that CNS disorders are the world’s leading cause of disability and necessitate more prolonged care and hospitalizations than almost all other diseases [5]. In particular, many CNS pathologies, such as neurodegenerative and enzymatic disorders (e.g. Parkinson’s, amyotrophic lateral sclerosis and Mucopolysaccharidosis), as well as functional recovery after spinal cord injuries, may benefit from IT [1, 10–12]. IT is presently used for treatment of spasticity and chronic pain caused by multiple sclerosis and cancer [3, 4]. IT systems, also known as pain pumps, consist of a pump that is surgically placed beneath the skin. The pump contains a medication that is released into the CSF via a flexible catheter [4]. For these patients, a bolus injection trial, whose injection is performed for approximately 1 minute, is used to test patient tolerance to the administered drug [13]. Another application of IT with several ongoing experimental trials is gene therapy, a procedure in which gene vectors are delivered and distributed to the CNS tissue via the CSF [14, 15].

IT is affected by several parameters, many of which are little understood. These parameters are as follows: 1. Solute baricity and chemical properties; 2. Catheter type, placement and orientation; 3. Infusion flow rate, volume and concentration; 4. Patient characteristics [16]. Additional complex biophysical aspects that affect the IT outcome include drug advection and diffusion within the CSF [17], absorption across the arachnoid membrane and interstitial penetration within the tissue [5]. At present, optimal IT protocols are not yet established. Therapy control is needed to provide an adequate therapeutic effect while minimizing possible risks, complications (e.g. catheter tip granuloma) and costs [17, 18]. In particular, gene therapy drugs can cost as much as 1 million USD per patient [19].

Several clinical and experimental studies have been performed to investigate the intrathecal drug distribution within the CSF. Experimental studies on non-human primates were carried out to investigate transport and tissue penetration mechanisms [1, 11], as well as pharmacokinetics and bioavailability [20], of the injected macromolecules. Papisov [1] found that drug and macromolecules delivery to the CNS can be pursued through the intrathecal route although several aspects affecting it, such as drug-cell interaction and CSF drainage, are not fully understood. Clinical studies in humans were performed in [21] to understand how continuous IT flow rates affect analgesia and observed that at a higher flow rate the patient’s pain feeling increased, a factor likely due to the increased drug dilution. An *in vitro* model was carried out in [22] to investigate the dependence of anaesthetic distribution on flow rate, catheter size and angle through and it found that all these parameters affect drug distribution and peak concentration. An *in vitro* model was also developed in [23] to investigate CSF dynamics around a catheter tip and it reported that steady streaming introduced by adjacent CSF vortices was the main driver of drug movement. Finally, a recent *in vitro* model was developed by Tangen et al. [24] to investigate the effects of the body position and lumbar drainage rates on the treatment of the subarachnoid hemorrhage.

Several numerical modeling studies have investigated drug transport in the spinal SAS. Myers [25] completed the first numerical model of IT in an idealized three-dimensional (3D), axisymmetric elliptic-shaped-geometry of the spinal SAS. This model did not include small anatomic features such as the spinal cord nerve roots, but it parametrically evaluated the influence of injection rate, catheter orientation and spinal-column size on drug distribution over a maximum period of five minutes. The authors found that low values of the ratio of the SAS and catheter cross-sectional dimensions produce more uniform drug distribution and that effects of catheter orientation are more pronounced at higher injection flow rates. More recently, [26] performed numerical simulations of lumbar IT in a 3D reconstruction of the spinal SAS (C1 to L2 levels) with approximated elliptical cross-sections and moving boundaries. The authors computed drug concentration in the CSF over a period of one hour after bolus injection and noticed that local drug distribution differences occurring between slow or bolus injection dissipate on a longer time scale. This model lacked a catheter geometry within the CSF and did not focus on the effects of specific injection parameters. Hsu [27] developed a two-dimensional model of the complete SAS (cerebral and spinal) and ventricles and showed that a 2X increase in CSF frequency and stroke volume enhanced drug dispersion and decreased peak concentration by 26 and 28%, respectively. This model also lacked small anatomic features within the CSF such as spinal cord nerve roots and arachnoid trabeculae. In another study, [28, 29], performed Lattice Boltzmann simulations (with periodic boundary conditions) on an elliptic SAS annulus that included idealized fine anatomical structures (i.e. nerves bundles and trabeculae) and showed that these anatomical structures produce CSF stirring effects and consequently enhance drug dispersion by a factor of 5 to 10. The effective longitudinal drug dispersion was found to be 1000 times higher than a reference molecular diffusivity. Finally, [30] showed that in models of the cervical SAS with NRDL, assuming CSF incompressibility and fixed SAS walls, the presence of microanatomical structures can speed up drug dispersion.

Despite the outstanding contributions available in literature, to the best of our knowledge, a 3D anatomically-detailed model has not been used to investigate the possibility and a range of effects of injection parameters on IT. We present a numerical model of catheter drug injection based on a patient-specific 3D geometry of the cervical SAS with idealized anatomical structures, namely dorsal and ventral spinal cord nerve rootlets and denticulate ligaments (NRDL).

## Materials and methods

We conducted a series of numerical simulations to investigate the impact of the following parameters on drug distribution within the CSF: (a) NRDL, (b) catheter position, (c) catheter angle and (d) injection flow rate. All simulations were carried out for a period up to 20 cardiac cycles (*T* = 0.78 *s* being the period), and drug injection was assumed to be continuous over that time span. Drug distribution was quantified in terms of drug peak concentration, axial spread, accumulation rate and appearance instant.

### Geometry and flow conditions

**Fig 1:**
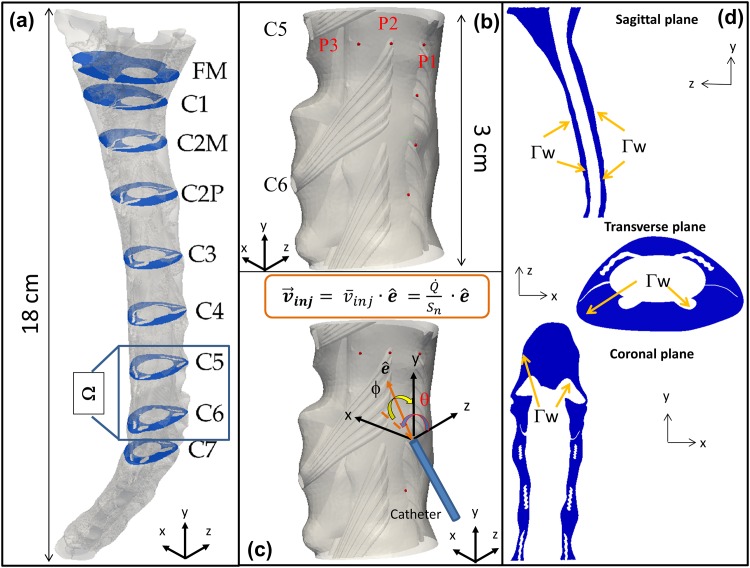
Geometry of the anatomical domain. (a) 3D geometry of the cervical SAS with NRDL also showing relevant anatomical cross sections; (b) Injection positions; (c) Spherical system to define catheter angle; (d) Illustrative sections of the cervical SAS: sagittal (x = 1.7 cm), transverse (y = 6 cm) and coronal (z = 0.85 cm).

We considered two different 3D geometries of the cervical SAS (ranging from the foramen magnum to ∼5 *cm* caudal to the seventh cervical vertebra (C7), as reported in Fig 1(a)) with rigid walls. A SAS geometry without NRDL (previously published by [31]) was obtained from manual segmentation of T2-weighted MRI sequences on a 22-years-old male healthy subject using ITK-Snap (Version 2.2, University of Pennsylvania). A more complex geometry of the SAS with detailed anatomical structures, was obtained by the addition of artificially constructed NRDL, based on anatomical ex-vivo measurements in the literature and reference to the MR images (further details are reported in [32]).

A patient-specific CSF flow rate, based on 4D Flow MRI measurements obtained for the same 22-years-old subject as above, was imposed at the flow inlet on the caudal end of the model (5 *cm* below the C7 level shown in Fig 1(a)). Furthermore, we applied no-slip boundary conditions at the SAS walls, a reference pressure value at the flow outlet on the cranial end of the model and a null-velocity as the initial condition.

Finally, to simulate drug infusion, we assumed that the characteristic size of the catheter tip (≈0.2 *mm* representative of the inner radius of a 22-gauge catheter) is small compared to that one of the SAS cross-section. Moreover, since we were not interested in assessing the effect of catheter tip penetration into the SAS, we assumed injection to occur in a tiny volume hereafter denoted by Ω_*n*_. In particular, Ω_*n*_ was defined by intersecting the SAS with a sphere having radius equal to the catheter inner radius and centered at the injection position on the arachnoid wall (specified below, when discussing the injection parameters). Hence, we enforced the injection velocity (**v**_**inj**_ in Fig 1(c)) through a Dirichlet boundary condition within Ω_*n*_. The injection direction was defined according to the angles *θ* and *ϕ* shown in Fig 1(c).

### Drug parameters

The injected drug was considered to be isobaric as in [27], with identical properties as water at body temperature having a kinematic viscosity and density of 7 ⋅ 10^−7^ *m*^2^/*s* and 1 ⋅ 10^3^ *kg*/*m*^3^, respectively. Moreover, molecular diffusion of the drug was not considered because typical values of drug diffusivity in the CSF are ∼10^−10^ − 10^−11^ *m*^2^/*s* [3, 33], rendering advection to be the dominant factor leading to drug spread. Furthermore, as observed by [30], diffusion has little affect on intrathecal drug dispersion. In addition, based on the considered short time window (∼ 15 s), we neglected drug absorption-desorption and degradation mechanisms that can play a major role on a longer time scale. By virtue of these positions, drug transport reduced to the transport of a passive scalar simply advected with the CSF.

### Injection simulations and parameters

The following numerical simulations, S_*i*_ (Table 1), were conducted to understand the impact of catheter position, angle, injection flow rate and CSF space anatomy:

1. S_1_-S_6_ to evaluate the effects of catheter position (perpendicular injection with a 6 *cm*/*s* injection speed);
2. S_2_, S_7_, S_8_ to evaluate the effects of catheter angle in a frontal position (injection at P_2_ with a 6 *cm*/*s* injection speed);
3. S_6_, S_9_, S_10_ to evaluate the effects of catheter angle in a lateral position (injection at P_6_ with a 6 *cm*/*s* injection speed);
4. S_2_, S_11_, S_12_ to evaluate the effects of injection speed (perpendicular injection at P_2_).

**Table 1:** Summary of injection simulations and parameters analyzed.

Test Case	Position	*θ*	*ϕ*	${\bar{v}}_{inj}\;\left[cm/s\right]$
S_1_	P_1_	0	0	6
S_2_	P_2_	0	0	6
S_3_	P_3_	0	0	6
S_4_	P_4_	0	0	6
S_5_	P_5_	0	0	6
S_6_	P_6_	0	0	6
S_7_	P_2_	0	45°	6
S_8_	P_2_	45°	0	6
S_9_	P_6_	0	45°	6
S_10_	P_6_	45°	0	6
S_11_	P_2_	0	0	3
S_12_	P_2_	0	0	0
${\text{S}}_{1}^{⋆}$	P_1_	0	0	6

*Position:* Six different injection positions were considered at the cervical level (between C_5_-C_7_ vertebra levels), located either on the dorsal (P_1_-P_4_) or on the dorsolateral side (P_5_-P_6_) of the spinal SAS, within the CSF close to the outer arachnoid wall, as shown in Fig 1(b). We located the catheter injection positions dorsally between consecutive nerve bundles, a location that is also accessible by a needle that can penetrate the intervertebral disks.

*Angle:* We considered three different catheter angles at a single fixed injection position. In particular, we assumed the injection jet perpendicular (*θ* = *ϕ* = 0°) to the spinal cord, inclined 45°-up (*θ* = 0°, *ϕ* = 45°) and 45°-right (*θ* = 45°, *ϕ* = 0°), where *θ* and *ϕ* are respectively the azimuth and elevation angles between the z-axis and the injection direction $\hat{e}$ shown in Fig 1(c).

*Flow:* We also investigated the effect of the injection speed (${\bar{v}}_{inj}$). The main value we adopted, i.e. ${\bar{v}}_{inj}=6\;cm/s$, was estimated as the ratio between a volumetric flow rate $\dot{Q}=0.5\;ml/min$ through a cross section *S*_*n*_ of a clinically used 22-gauge catheter [34–36]. This flow rate is also representative of the ongoing screening trial for intrathecal Baclofen administration [37–39]. Moreover, to account for slower injections, we also considered 3 and 0 *cm*/*s* (the latter value describes a limit case where the drug starts to be advected with the local fluid velocity at the injection point).

*Anatomy:* To investigate the impact of small anatomical structures on drug distribution, simulation ${\text{S}}_{1}^{⋆}$ was conducted with identical properties as S_1_ except for a geometry that did not include the NRDL. Two additional test cases were also conducted with the catheter removed from the model (no drug injected) with NRDL (test case PS_1_) and without NRDL (${\text{PS}}_{1}^{⋆}$). These test cases are not reported in Table 1 because they are not directly related to IT working parameters, yet the associated results are discussed.

### Governing equations

We considered that the CSF flows in the SAS domain because of time-varying pressure gradient generated by the cardiac pulsation. Moreover, since the CSF is a fluid similar to water, we assumed it to be incompressible, and we retained the viscous effects since they play a key role in a complex confined boundary like the SAS including NRDL. To obtain the CSF velocity in the SAS (below denoted by Ω), we thus adopted the following governing Eq (1) representing the mass and the momentum balance (Navier-Stokes equations) for the fluid:

$$\begin{array}{c} \left\{ \begin{array}{cc} \partial_{t}u+(u\cdot \nabla )u-\nu \Delta u+\frac{1}{\rho }\nabla p=0,\;\text{in} & \Omega \times T,\\ \nabla \cdot u=0,\;\text{in} & \Omega \times T,\\ {u|}_{\Gamma_{i}}=(0,v_{in}\left(t\right),0),\;\text{in} & \Gamma_{i}\times T,\\ {u|}_{\Gamma_{w}}=0,\;\text{in} & \Gamma_{w}\times T,\\ {u|}_{\Omega_{n}}=v_{\text{inj}},\;\text{in} & \Omega_{n}\times T,\\ {p|}_{\Gamma_{o}}=0,\;\text{in} & \Gamma_{o}\times T,\\ u=0,\;\text{in} & \Omega \times \{t=0\}, \end{array}\right. \end{array}$$ (1)

where **u** = (*u*(**x**, *t*), *v*(**x**, *t*), *w*(**x**, *t*)) and *p* = *p*(**x**, *t*) are the unknowns of the problem and respectively denote the CSF velocity and pressure (**x** = (*x*, *y*, *z*) is the coordinate vector). Moreover, T denotes a chosen time-interval, *ν* and *ρ* respectively represent the CSF kinematic viscosity and density (*ν* = 7 ⋅ 10^−7^ *m*^2^/*s*, *ρ* = 10^3^ *kg*/*m*^3^ as for water at 37°), and *v*_*in*_ represents the y-component of the CSF velocity at the inflow (Γ_*i*_) cross-section, obtained from experimental measurements. Furthermore, **v**_**inj**_ denotes the prescribed injection velocity (directly assigned within the injection volume Ω_*n*_). Finally, Γ_*w*_ represents the SAS walls, shown in Fig 1(d), and Γ_*o*_ the outflow cross-section on the cranial end of the model.

We studied drug distribution by adopting a Lagrangian particle tracking approach (see e.g. [40]). Conversely to the Eulerian continuum approach, we described the drug through *p* discrete particles (massless, thanks to the isobaric approximation) that do not collide with one another, and we solved the momentum equation for each particle at every time step. The vector $\bar{\xi }=\left(x_{1},x_{2},\cdots ,x_{p}\right)$, where $x_{i}\in R^{3}$ (*i* = (1, 2, ⋯, p)) is the unknown position of the i-th particle, identified the drug distribution. We determined the particle pathline by integration of the following ordinary differential equation:

$$\begin{array}{c} \left\{ \begin{array}{cc} \frac{dx_{i}}{dt} & =u\left(x_{i},t\right),\;i=\left(1,2,\cdots ,p\right),\\ x_{i}\left(t_{0}\right) & =x_{i0}, \end{array}\right. \end{array}$$ (2)

which represents the advective transport coupled with a specific initial condition. Moreover, with regard to particle seeding, we injected a fixed number of particles per time step (${\dot{N}}_{p}$) at random points inside a sphere having radius equal to the catheter inner radius and located adjacent to the injection position (the coordinates of the sphere center were obtained translating the coordinates of the injection position by a quantity equal to the sphere radius).

The numerical methods adopted in order to integrate the considered governing equations are detailed in S1 Appendix; related independence studies are detailed in S2 Appendix.

Hereafter, unless differently specified, we show the concentration *c* that denotes a normalized drug concentration on a fixed cross-section at a distance of Δ*y* from the injection point. In particular, we divided the concentration in a thin volume slice (0.8 *mm* thick) centered at the considered cross-section by the total average concentration in the whole domain after 10 cycles.

## Results

### Effects of the nerve roots and denticulate ligaments

**Fig 2:**
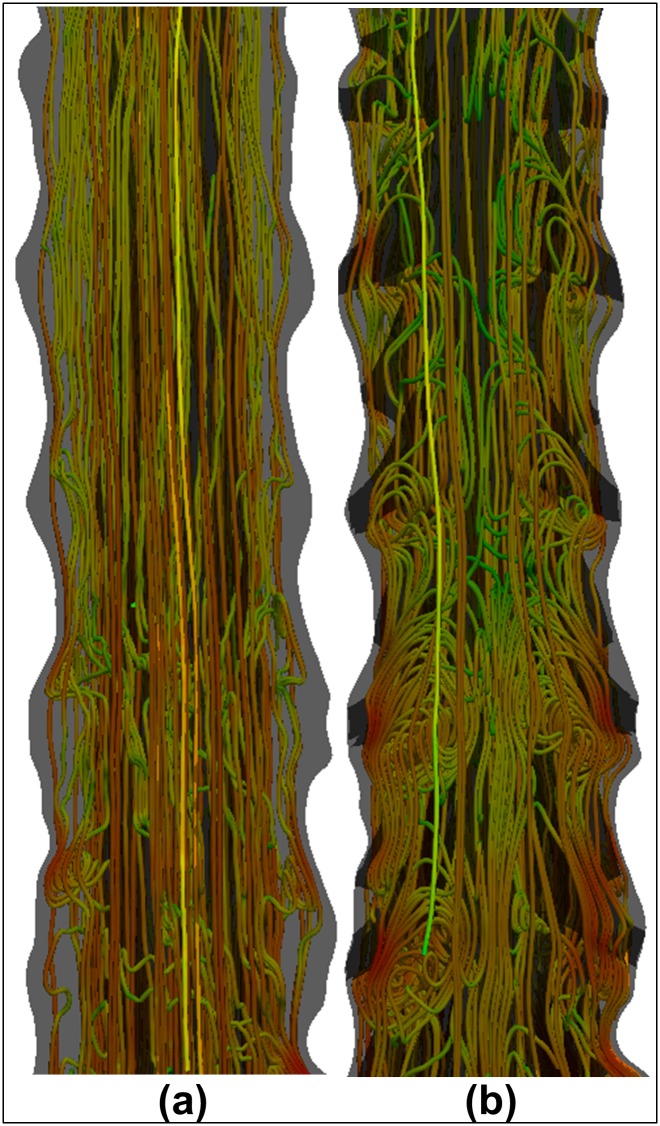
CSF flow streamlines at t/T = 4.5. The reported streamlines are obtained from test cases (a) ${\text{PS}}_{1}^{⋆}$ (without NRDL) and (b) PS_1_ (with NRDL).

**Fig 3:**
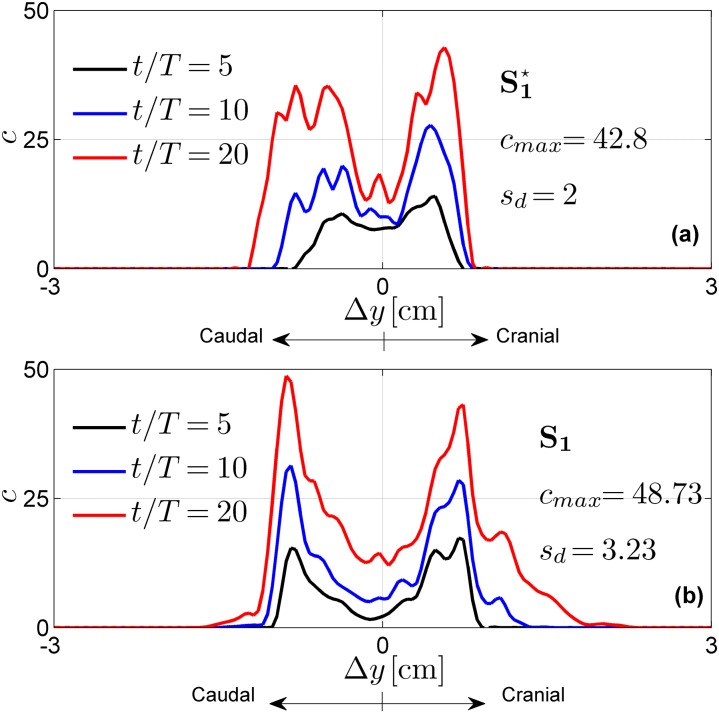
Normalized drug concentration profiles as a function of the distance Δ*y* from the injection point P_1_. The reported profiles result from (a) ${\text{S}}_{1}^{⋆}$ and (b) S_1_.

**Fig 4:**
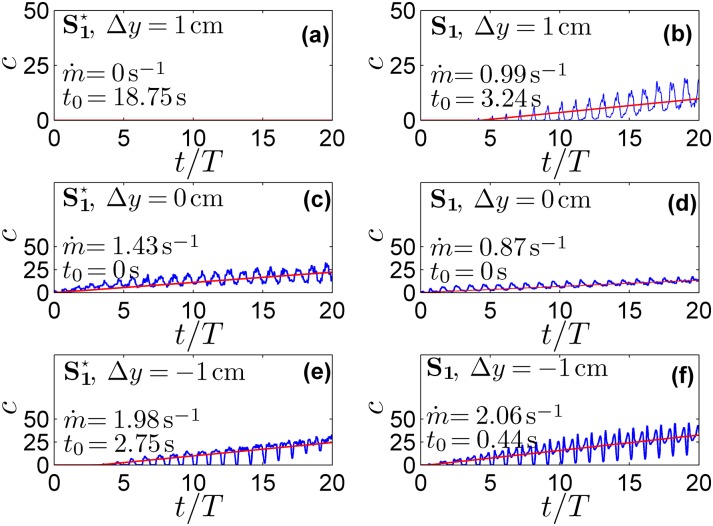
Time evolution of the normalized drug concentration and relative linear fitting. The blue lines represent the time evolution of the normalized drug concentration at different cross sections from the injection point for (a) ${\text{S}}_{1}^{⋆}$ and (b) S_1_. The red line indicates the relative linear fitting ($\bar{c}$).

The effects of NRDL on the CSF fluid flow are shown in Figs 2–4 and Table 2. In particular, Fig 2 shows the CSF streamlines at t = 4.5 T for test cases PS_1_ (with NRDL) and ${\text{PS}}_{1}^{⋆}$ (without NRDL). In addition, Table 2 shows the CSF peak velocity (clinically called systolic velocity, *v*_*sys*_) and the peak pressure drop between two consecutive spinal cross-sections (Δ*p*_*peak*_) selected out of those reported in Fig 1(a).

**Table 2:** Hydrodynamics parameters within each spinal segment resulting from test cases PS_1_ and ${\text{PS}}_{1}^{⋆}$.

Level	*v*_*sys*_[*cm*/*s*]		Δ*p*_*peak*_[*Pa*]	
	*PS*_1_	$PS_{1}^{⋆}$	*PS*_1_	$PS_{1}^{⋆}$
FM	0.98	0.93	-	-
C1	2.09	1.60	0.87	0.80
C2M	2.71	1.67	4.15	3.33
C2P	2.29	1.46	2.37	2.00
C3	3.85	2.26	6.20	5.11
C4	3.93	2.51	6.89	5.42
C5	4.56	2.76	7.80	5.44
C6	4.44	2.47	8.39	6.12
C7	5.57	4.46	6.61	4.73

The strong differences in the CSF flow field caused by the NRDL microanatomy (i.e. microanatomy-induced CSF mixing effects) were expected to alter drug transport. This was verified by the results shown in Figs 3 and 4, where the former reports the concentration at fixed times versus Δ*y* and the latter shows the concentration at selected Δ*y* versus time. Moreover, from the spatial distribution at a fixed instant, we extracted the maximum drug concentration (*c*_*max*_) at the selected injection time and the corresponding extent of drug spreading (*s*_*d*_) along the y-axis. After a linear average growth was observed in Fig 4, we introduced the following linear regression:

$$\begin{array}{c} \bar{c}\left(\Delta y,t\right)=\dot{m}\;(t-t_{0}\left(\Delta y\right)),\;\;\text{for}\;\;t\geq t_{0}, \end{array}$$

so as to extract the drug accumulation rate $\dot{m}$ and the time *t*_0_ when drug first appears at each section.

In light of the noticeable differences brought by NRDL on the underlying CSF flow field and, consequently, on drug distribution, we analyzed the effects of the injection parameters only in relation to the mesh with NRDL, so as to obtain more realistic results.

### Effects of the injection parameters

**Fig 5:**
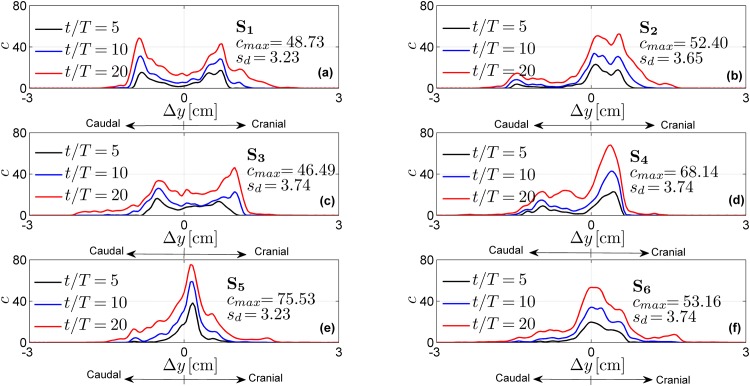
Normalized drug concentration profiles as a function of the distance Δ*y* from the injection point for different injection positions. The profiles are obtained with test cases S_1_-S_6_.

**Fig 6:**
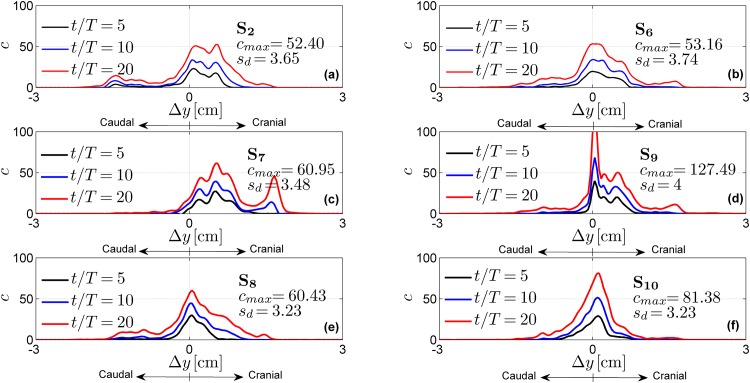
Normalized drug concentration profiles as a function of the distance Δ*y* from the injection point for different catheter angles. The profiles are obtained with a dorsal injection located at P_2_ (for test cases S_2_, S_7_, S_8_) and with a lateral injection located at P_6_ (for test cases S_6_, S_9_, S_10_).

**Fig 7:**
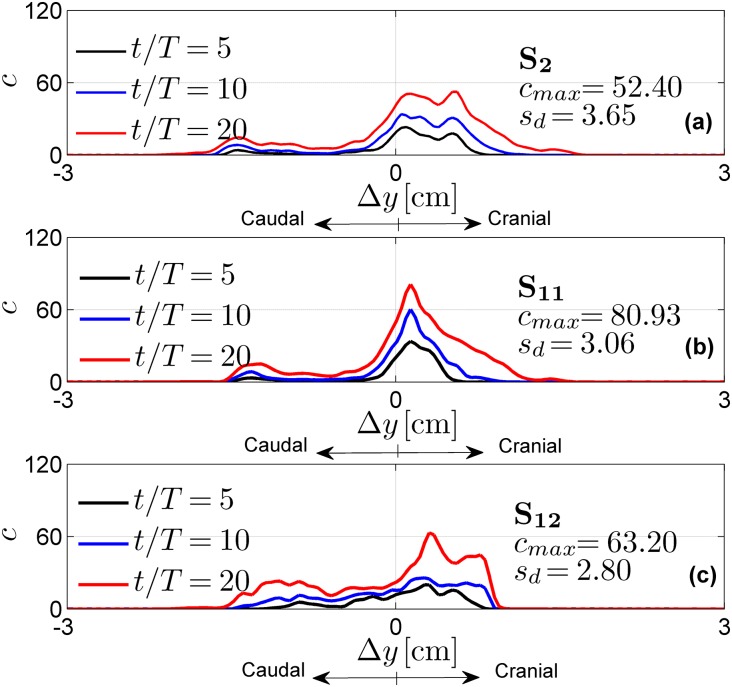
Normalized drug concentration profiles as a function of the distance Δ*y* from the injection point P_2_ for different injection speeds. The profiles are obtained with test cases S_2_, S_11_, S_12_.

**Fig 8:**
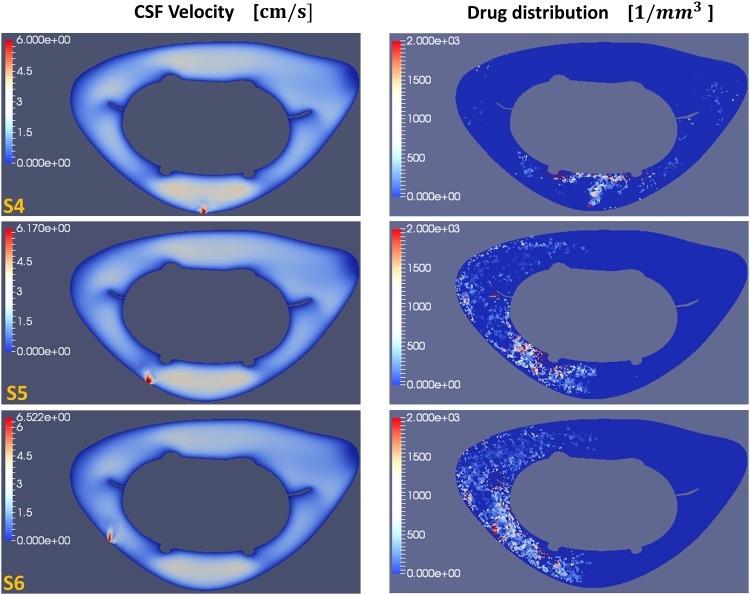
Cross-sectional views of the CSF velocity magnitude and dimensional drug concentration. (left) Magnitude of the CSF velocity at *t* = 20 *T* and (right) corresponding dimensional drug concentration (number of particles per volume) at Δ*y* = 0 *cm* (C5-level) for test cases S_4_, S_5_ and S_6_.

The effects of the injection parameters on the drug spatial distributions are shown in Figs 5–7 for all test cases. These figures also report maximum concentration *c*_*max*_ and drug spread *s*_*d*_ at injection time *t* = 20 *T*. Tables 3–5 report the drug accumulation rate ($\dot{m}$) and appearance time (*t*_0_) for selected values of Δ*y*. In particular, Table 3 reports the effects of the injection position, Table 4 the effects of catheter angles and Table 5 the effects of injection speed. Finally, in Fig 8 we report cross-sectional views of the CSF velocity and of the dimensional drug concentration (number of particles per *mm*^3^) at *t* = 20 *T*, to qualitatively visualize how the drug spreads in the SAS annulus while moving the injection position laterally.

**Table 3:** Drug accumulation rate (1/s) and appearance instant (s), as obtained by varying the catheter position.

Δ*y*[*cm*]	S_1_		S_2_		S_3_		S_4_		S_5_		S_6_	
	$\dot{m}$	*t*_0_	$\dot{m}$	*t*_0_	$\dot{m}$	*t*_0_	$\dot{m}$	*t*_0_	$\dot{m}$	*t*_0_	$\dot{m}$	*t*_0_
1	0.99	3.24	0.52	2.46	1.13	2.46	0.09	6.33	0.27	3.18	0.39	3.12
2/3	3.24	0.87	1.51	0.87	2.46	0.84	0.49	1.68	0.63	1.72	0.76	1.65
1/3	1.63	0.72	2.42	0.75	1.13	0.72	2.09	0.78	1.82	0.78	1.97	0.75
0	0.87	0.0	2.87	0.0	1.19	0.0	2.79	0.0	3.21	0.0	2.65	0.0
-1/3	0.84	0.22	1.42	0.22	1.30	0.22	1.30	0.22	2.23	0.22	2.17	0.22
-2/3	1.58	0.31	0.43	0.31	1.87	0.31	1.22	0.31	1.58	0.31	1.10	0.31
-1	2.06	0.44	0.41	0.37	0.87	1.22	0.31	0.37	0.70	1.12	0.72	1.19

**Table 4:** Drug accumulation rate (1/s) and appearance instant (s), as obtained by varying the catheter angle.

Δ*y*[*cm*]	S_2_		S_7_		S_8_		S_6_		S_9_		S_10_	
	$\dot{m}$	*t*_0_	$\dot{m}$	*t*_0_	$\dot{m}$	*t*_0_	$\dot{m}$	*t*_0_	$\dot{m}$	*t*_0_	$\dot{m}$	*t*_0_
1	0.52	2.46	0.71	0.90	0.68	3.99	0.30	3.12	0.56	2.43	0.14	2.46
2/3	1.51	0.87	1.57	0.81	1.36	0.90	0.59	1.65	0.99	0.84	0.35	1.59
1/3	2.42	0.75	2.79	0.66	1.86	0.78	1.52	0.75	2.55	0.66	1.49	0.75
0	2.87	0.0	2.26	0.0	2.58	0.0	2.04	0.0	7.66	0.0	3.79	0.0
-1/3	1.42	0.22	0.65	0.28	2.13	0.22	1.66	0.22	1.40	0.28	2.89	0.22
-2/3	0.43	0.31	0.15	0.41	0.69	0.31	0.84	0.31	0.56	0.44	1.55	0.34
-1	0.41	0.37	0.08	5.05	0.60	0.41	0.56	1.19	0.51	2.75	0.53	1.90

**Table 5:** Drug accumulation rate (1/s) and appearance instant (s), as obtained by varying the injection speed.

Δ*y*[*cm*]	*S*_2_		S_11_		S_12_	
	$\dot{m}$	*t*_0_	$\dot{m}$	*t*_0_	$\dot{m}$	*t*_0_
1	0.52	2.46	0.42	4.84	0.24	4.80
2/3	1.51	0.87	1.39	1.68	0.91	3.21
1/3	2.42	0.75	2.15	0.81	2.47	0.81
0	2.87	0.0	3.52	0.0	2.29	0.0
-1/3	1.42	0.22	1.46	0.25	0.85	0.25
-2/3	0.43	0.31	0.37	0.31	1.15	0.37
-1	0.41	0.37	0.41	0.41	0.74	1.22

## Discussion

This study applied computational fluid dynamics to assess IT in an anatomically detailed model of the upper cervical spine. Overall, our findings suggest that IT is sensitive to many factors and that computational modeling can offer insight into how individual factors may be tuned to produce a desired drug delivery profile. Our approach was to model the drug as massless particles within the flow field injected from a catheter located in the cervical spine and parametrically assess the impact of the following parameters on the drug spread over a short time scale (∼ 15 s) following the injection: a) influence of fine anatomical structures, b) catheter location, c) catheter angle, d) catheter flow rate. The key findings of this study were the following:

1. Small anatomic structures (NRDL) within the SAS increase axial drug spread by 60% in comparison to a model without these structures;
2. Catheter injection location can alter the axial drug distribution up to ∼90%;
3. Catheter angle can steer/shift the axial drug distribution up to ∼90%;
4. Injection flow rate modulates the peak magnitude of drug concentration up to ∼78%. Also, catheter flow rate can alter the axial distribution.

Herein, we describe the above findings in context of the results and compare the findings to previous studies focused on IT.

### Spinal cord nerve roots and denticulate ligaments (NRDL) increase drug mixing

The presence of NRDL within the computational model was found to increase drug spread by 60% and alter the axial distribution of the drug along the SAS. The preliminary CFD simulations on mesh no.1 and no.2 without injection (test cases ${\text{PS}}_{1}^{⋆}$ and PS_1_, respectively), showed that micro-anatomical structures induce vorticity (Fig 2), and NRDL generate a complex CSF flow field characterized by vortices aligned along the SAS axis that greatly enhance mixing through mechanisms similar to those observed in a turbulent flow. These results are consistent with previous studies [28, 30, 32]. Moreover, we observed that the mixing phenomenon occurs when CSF velocity shifts from the cranial to the caudal direction and vice versa, as shown in Fig 2(b) for *t*/*T* = 4.5.

Furthermore, we also noticed that microanatomy increases the CSF systolic velocity and peak pressure drop. In particular, as reported in Table 2, we observed a total average increase of 29% for velocity and 21% for pressure. Considering some deviation due to the specific spinal levels selected to observe the solution, these results are consistent with those reported in [32], where the authors ran simulations on the same geometry using ANSYS Fluent. Finally, the pressure drop between the FM and C4 is similar in magnitude with results obtained by [30] although the velocity they reported was three times lower. This elevated pressure drop is likely attributable to the presence of arachnoid trabeculae included in [30], an anatomic feature that was not included in the model used in the current study. [41, 42] completed a study of CSF flow around the brain that included arachnoid trabeculae and these structures have been shown to increase pressure gradients by a factor that varies according to their density and size. Also, the geometric and flow boundary conditions used by [30] were different from the present study. Thus, further simulations would be necessary to carry out a detailed comparison of results.

The CSF stirring effects due to NRDL impact drug transport is shown in Figs 3 and 4. In particular, Fig 3 shows that for the mesh with NRDL the drug spreads much farther, both in the cranial and caudal direction. Indeed, *s*_*d*_ is approximately 60% higher than in the case without NRDL. This is confirmed by the parameters $\dot{m}$ and *t*_0_ assessed through linear fitting shown in Fig 4. In particular, for the case with NRDL, *t*_0_ assumes lower values, especially farther from the injection point, indicating a faster drug spread. At Δ*y* = ±1 *cm*, *t*_0_ is ∼6 times lower than the corresponding parameter for the case without NRDL. This aspect is further confirmed by the value of the accumulation term $\dot{m}$ at the injection cross-section (Δ*y* = 0 *cm*). Indeed, it is 35% lower than the case without NRDL, showing that drug accumulates less around the injection point and moves farther (e.g. in the case without NRDL drug does not reach the cross-section at Δ*y* = 1 *cm*).

### Catheter injection location alters axial drug distribution and spread rate

Our results showed that catheter injection location can alter the axial distribution and spread rate significantly. This IT effect is likely attributable to locally elevated CSF velocities, or jets, that may occur between NRDL and within other constricted SAS spaces. In particular, with regard to Fig 5, we observe two different trends when the catheter is inserted in the dorsal SAS (P_1_-P_4_). For an injection at P1 and P3, located in the middle of the nerve bundle, drug distributes symmetrically with two peaks in the caudal and cranial directions. For an injection at P_2_ and P_4_, located between two consecutive nerve bundles, the drug spreads with a single peak located in the cranial direction and a uniform tail distributed caudally.

These trends can be justified in light of the CSF velocity profile at these spinal levels. We observed velocity jets in P_1_ and P_3_ due to restriction of the SAS at those locations (velocity profile modulations caused by domain restrictions are also analyzed for idealized geometries, e.g. in [43]). Indeed, for an injection at P1 and P3 we do not observe concentration peaks around the injection site (where instead the drug is carried away quickly). In contrast, they appear for drug injection at P_2_ and P_4_. Thus, the different drug distributions reflect geometry-induced flow patterns. The characteristic peaks of each distribution can be also detected by the higher values of $\dot{m}$ reported in Table 3. Finally, from the same table we can also observe that all the aforementioned injection positions make the drug spread faster caudally than cranially (*t*_0_ assumes lower values for Δ*y* < 0).

For an injection performed at the dorsolateral position, namely P5 and P6, we notice that drug distribution is similar to a bell profile. The corresponding values for $\dot{m}$ and *t*_0_ in Table 3, decrease and increase, respectively along both directions starting from the injection position.

Similar to our results, [26] observed that the drug spreads cranially in the ventral and dorsal SAS, while it moves caudally in the lateral region. Moreover, Fig 8 shows that shifting the injection at lateral positions, the drug starts to spread around the spinal cord, and by injecting at P_5_ and P_6_ (the two most lateral positions) it moves towards the ventral side of the SAS (Fig 8(b) and 8(c)). Finally, from Fig 5 it can be seen that although different injections positions do not produce large differences in terms of drug spreading (*s*_*d*_), they have significant impact on the shape of the drug distribution. All injection positions (with perpendicular catheter angle) result in a faster drug spreading in the caudal direction. However, even if the peak concentration generally occurs in the cranial direction, based on the limited number of considered test-cases it is hard to extract a general conclusion.

### Catheter angle can tune drug spread direction

The effects of catheter angle are shown in Fig 6 respectively at a fixed dorsal (Fig 6(a), 6(c) and 6(e)) and dorsolateral (Fig 6(b), 6(d) and 6(f)) injection position. In contrast to catheter position, catheter angle was not found to broadly modify the shape of the drug concentration profile, but did shift the distribution. In particular, among the evaluated angles, the upward angle (test cases S_7_ and S_9_) moved the drug more cranially, as expected. This is confirmed by parameters listed in Table 4 where the upward injections produce the highest values of $\dot{m}$ at the cranial levels and the lowest at the caudal levels compared to the results obtained with different angles in the same position. Consistently, S_7_ and S_9_ show the lowest cranial values of *t*_0_ and the highest caudal ones. Therefore, it means that the drug spreads faster in the head direction where it accumulates.

Conversely, the right injection in the dorsal position (S_8_) shifted the peak concentration caudally, increasing the accumulation in the caudal direction. Indeed, between the test cases performed with dorsal injection and reported in Table 4 (i.e. S_2_, S_7_ and S_8_), S_8_ gives the highest values of *t*_0_ for Δ*y* > 0 and of $\dot{m}$ for Δ*y* < 0. This result is consistent with that reported by [25], which shows an increment of caudal-to-cranial drug concentration ratio with a catheter angle towards the SAS wall. However, this aspect is less evident with the rightward angle at a lateral injection position (S_10_). Indeed, we can only notice higher values of the accumulation term $\dot{m}$ for Δ*y* < 0 compared to that of S_6_ and S_9_. However, from Fig 6 we notice that the rightward injection, in both dorsal and lateral position, reduces the drug spread *s*_*d*_. Finally, the effects of different catheter angles are more pronounced in terms of variations of peak concentration and drug spread by lateral injection position (e.g. S_9_ provides a peak concentration around 130 that is more than twice the one of S_6_).

### Drug injection flow rate alters peak drug concentration

Drug injection flow rate altered *c*_*max*_ and *s*_*d*_ (Fig 7). An higher injection speed increased drug spread and reduced the peak concentration. These results are consistent with experimental observations by [22]. Moreover, this trend is also consistent with the results in Fig 7(c) for longer injection times. Indeed, the null injection speed, S_12_ represents a limit case where concentration is entirely relying on the underlying CSF flow field (for which it might take time for local maxima to appear). Finally, the enhanced drug spread obtained at higher flow rate can be appreciated in Table 5 that reports for S_12_ values of *t*_0_ in the cranial and caudal part respectively ∼ twice and four times the ones of S_2_.

## Limitations

Our approach included a relatively small axial section of the upper spine (18 cm axial length) within the computational domain. The limited domain extension did not allow us to take into consideration drug delivery to the brain, which represents an important goal of IT therapy, as well as drug injection within the lumbar spine (often performed in IT protocol). However, our focus was on the cervical spine as this region has not been specifically examined in the literature and is of clinical interest for certain drug delivery protocols [44–46], gene therapy [14] and for targeting drug delivery to the cord or nerve roots. In addition, the computational geometry did not include arachnoid trabeculae, structures that have been shown in the past to alter the CSF flow field [28, 30, 42]. The proposed modeling framework can be used to further investigate the impact of arachnoid trabeculae in future studies. However, we expect the key findings of the present study, using variational analysis by altering specific factors, to hold even if small anatomic structures are included. Finally, we did not lose any particles through the open domain boundaries, since none of them reached the upper/lower ends of the computational domain, yet adopting a full CSF space geometry characterized by closed boundaries would improve future CNS studies.

The complex SAS geometry requires CPU-demanding simulations since we adopted a forward-modeling approach to resolve the physics both in space and time. Thus, we chose to simulate a limited time window (15 s). Each simulation required a total of 960 CPU hours, with a total of 12700 CPU hours for all simulations performed in the study. The considered time window did not allow examination of bolus versus continuous IT over a longer time window, although our model can also describe the effects of discontinuous injection, as shown by preliminary numerical experiments (cf. S3 Appendix). In addition, the large CPU time restricted the number of the injection parameters we were able to analyze.

It should be noted that on a longer time scale, additional physical effects, such as drug diffusion and extravasation, must also be more carefully considered. It is therefore important to extend the simulation time window to improve clinical relevance of the numerical model. To reach this goal, the numerical schemes must be improved to reduce computational cost. Further model refinements include the adoption of non isobaric drug, since baricity plays an important role in IT [47], as well as reaction terms in the drug transport equation for modeling the physical interaction with porous tissue adjacent to the SAS membranes [48]. Specific drug species as well as finer interaction mechanisms/dynamics are important aspects to be included in future studies so to properly plan IT treatments on a longer time scale for a particular drug. However, it is worth highlighting that specific pharmacokinetics/dynamics parameters are not often available or easily detectable in a closed domain such as the lumbar SAS region. In addition, the breathing force affecting the CSF flow field through the mobility of the SAS walls may be important. A study by Cheng found that the effect of breathing on the CSF flow may not be important [49]. Other studies have indicated that CSF flow can be altered by respiration [50]. Future work could help understand the clinical relevance of different drug distributions by coupling in vivo MRI measurements of CSF flow, geometry and drug spread, and by improving the provided computational framework with a more extensive anatomical domain, up to including specific species transport equations to suitably describe transport mechanisms on a longer time scale.

Finally, the specific catheter geometry and drug velocity profile when exiting the catheter should be introduced to investigate possible steady streaming effects around the catheter that may enhance drug dispersion (see e.g. [23]). In general, however, experimental data related to cervical injection (in particular over a short time window) are needed in order to fully validate the numerical results.

## Conclusions

We addressed the IT treatment within the cervical spine and the role of injection parameters, namely the catheter position, angle and injection flow rate on drug distribution. Within our model based on known physical laws, all investigated parameters were found to impact the IT distribution.

Catheter position was found to affect the drug distribution profile. Catheter angle shifted the concentration profile along the spine. Higher injection flow rates enhanced drug spread while reducing the peak concentration.

The computational modeling approach provides detailed insight into how the drug concentration is impacted within the CSF. The threshold at which these alterations would make a clinically significant difference is not yet known. We cannot extract general working guidelines based on these results, which however suggest to choose the injection position in view of the targeted therapeutic area. Future work could help understand the clinical relevance of different drug distributions by coupling in vivo MRI measurements of CSF flow, geometry and drug spread, by improving the provided computational framework with a more extensive anatomical domain, detailed drug properties and transport mechanisms on a longer time frame.

## Supporting information

S1 Appendix Numerical methods. Description of the numerical approach adopted to carry out the performed simulations.
(PDF)

S2 Appendix Independence studies. Convergence studies of the shown solutions.
(PDF)

S3 Appendix Concentration trend after injection. Exemplary numerical results showing drug concentration evolution after injection for test case S_1_.
(PDF)
