# Supplementary material for: A numerical investigation of intrathecal isobaric drug dispersion within the cervical subarachnoid space
Source: PLoS One. 2017 Mar 15;12(3):e0173680. doi: 10.1371/journal.pone.0173680 (PMC5351861; doi:10.1371/journal.pone.0173680)
Supplement: S1 Appendix — Description of the numerical approach adopted to carry out the performed simulations. (PDF) [file pone.0173680.s001.pdf]

Supporting Information S1

A Numerical Investigation of Intrathecal Isobaric Drug Dispersion within the Cervical Subarachnoid Space

P.T. Haga[1◑], G. Pizzichelli[2,3◑], M. Mortensen[1,4¤], M. Kuchta[4], S. Heidari Pahlavian[5], E. Sinibaldi[2], B.A. Martin[6*], K.A. Mardal[1,4]

1 Center for Biomedical Computing, Simula Research Laboratory, Fornebu, Norway
2 Istituto Italiano di Tecnologia, Center for Micro-BioRobotics, Pontedera, Italy
3 Scuola Superiore Sant'Anna, The BioRobotics Institute, Pontedera, Italy
4 Dept. of Mathematics, University of Oslo, Oslo, Norway
5 Conquer Chiari Research Center, Dept. of Mech. Engineering, University of Akron, Akron, Ohio, USA
6 Dept. of Biological Engineering, The University of Idaho, 875 Perimeter Drive MS 0904, Moscow, Idaho, USA

◑These authors contributed equally to this work.
* Corresponding author: brynm@uidaho.edu

Numerical methods

We performed finite element method simulations in FEniCS [1]. We generated nonuniform unstructured computational meshes also using ANSYS ICEM CFD (ANSYS Inc., Canonsburg, PA): two meshes with tetrahedral elements, having 1.7 million elements and 0.3 million nodes for the geometry without NRDL, and 11.3 million elements and 2.2 million nodes for the geometry with NRDL.

The Navier-Stokes problem and particle tracking problem were solved in succession using the Open Source Navier Stokes solver *Oasis*, a high-level/high-performance solver using Python interfaced with FEniCS, designed to minimize numerical dissipation and capture transitional effects [2]. An efficient implementation of the Lagrangian particle tracking solver for FEniCS was used that allowed a fast computation of each trajectory (https://github.com/MiroK/lagrangian-particles.git).

The Navier-Stokes equation was integrated by a non-iterative fractional step method (with time step $\Delta t = T/250$) with regular linear (P1-P1) Lagrangian finite elements for both velocity and pressure. The particle transport problem was then advanced in time through a 2nd order Runge-Kutta method (1 correction step) and the same time step adopted above. Concentration was obtained by adopting P0 finite elements (for each element concentration was computed by considering the number of particles it contained). For completeness, let us mention that our Lagrangian tracker did not lose any particles through the open domain boundaries, since none of them reached the upper/lower ends of the computational domain. It only lost 0.04% of the particles (i.e. 54 particles), in particular close to the pia (cord boundary) opposite the injection position, because they traveled farther than one cell per time-step.

We performed simulations on the Abel supercomputer at the University of Oslo, using MPI for parallel performance, with 96 CPU cores (960 CPU h) when adopting the mesh with NRDL and 16 CPU cores (110 CPU h) for the mesh without NRDL.

References

1. Logg A, Mardal KA, Wells GN. Automated solution of differential equations by the finite element method: The FEniCS book. Springer Science & Business Media; 2012.

2. Mortensen M, Valen-Sendstad K. Oasis: A high level/high-performance open source navier–stokes solver. Comput Phys Commun. 2015;188(0):177–188.
