# Supplementary material for: A numerical investigation of intrathecal isobaric drug dispersion within the cervical subarachnoid space
Source: PLoS One. 2017 Mar 15;12(3):e0173680. doi: 10.1371/journal.pone.0173680 (PMC5351861; doi:10.1371/journal.pone.0173680)
Supplement: S2 Appendix — Convergence studies of the shown solutions. (PDF) [file pone.0173680.s002.pdf]

Supporting Information S2

A Numerical Investigation of Intrathecal Isobaric Drug Dispersion within the Cervical Subarachnoid Space

P.T. Haga[1◑], G. Pizzichelli[2,3◑], M. Mortensen[1,4¤], M. Kuchta[4], S. Heidari Pahlavian[5], E. Sinibaldi[2], B.A. Martin[6*], K.A. Mardal[1,4]

1 Center for Biomedical Computing, Simula Research Laboratory, Fornebu, Norway
2 Istituto Italiano di Tecnologia, Center for Micro-BioRobotics, Pontedera, Italy
3 Scuola Superiore Sant'Anna, The BioRobotics Institute, Pontedera, Italy
4 Dept. of Mathematics, University of Oslo, Oslo, Norway
5 Conquer Chiari Research Center, Dept. of Mech. Engineering, University of Akron, Akron, Ohio, USA
6 Dept. of Biological Engineering, The University of Idaho, 875 Perimeter Drive MS 0904, Moscow, Idaho, USA

◑These authors contributed equally to this work.
* Corresponding author: brynm@uidaho.edu

Independence studies

Preliminary investigations for the convergence of p and $\mathbf{u}$, showed that these unknowns were less sensitive to the grid and time resolution than the concentration [1]. In light of that finding we report the independence studies from the time step, grid size and number of injected particles only for the concentration profile.

Representative results concerning the drug concentration are reported in Fig.1, where c represents a normalized drug concentration on a fixed cross-section located $1\,cm$ below the injection point. In particular, we divided the concentration in a thin volume slice ($0.8\,mm$ thick) centered at the considered cross-section by the total average concentration in the whole domain after 10 cycles. The discrepancy between two subsequent solutions was obtained by refining the discretization (by separately considering mesh size, time-step and number of injected particles): c_c and c_f were defined as the previous (coarser) and the subsequent (finer) discretization, and we quantified the discrepancy through the following L_2-norm:

$$e_{\%}(\Delta y) = \frac{(\int_T |c_f^2(\Delta y, t) - c_c^2(\Delta y, t)|\, dt)^{1/2}}{(\int_T c_f^2(\Delta y, t)\, dt)^{1/2}} \times 100.$$

We assessed time-step independence by carrying out the computations over twenty periods using time-step sizes of T/250, T/500 and T/1000 (all satisfying the CFL condition for solver stability). The relative error between the solutions obtained with time-step $\Delta t = T/250$ and $T/500$ was below 2%, while the one between $\Delta t = T/500$ and $T/1000$ was $\approx 1\%$, so that in all simulations we adopted the time-step $\Delta t = T/250$ (Fig.1(a)).

To evaluate grid size independence we generated a finer version of the NRDL mesh with 37 million cells. The results obtained with the two different mesh resolutions are

reported in Fig.1(b). Since we obtained a relative error below 3%, we used the coarser mesh for the simulations.

Finally, we assessed the independence from the number of injected particles (see Fig.1(c)). The relative error when passing from $\dot{N}_p = 20$ to $\dot{N}_p = 60$ was below 0.8%, while the one obtained by passing from $\dot{N}_p = 5$ to $\dot{N}_p = 20$ was around 2%. We adopted $\dot{N}_p = 20$ to enhance the computation of drug concentration by volume averaging.

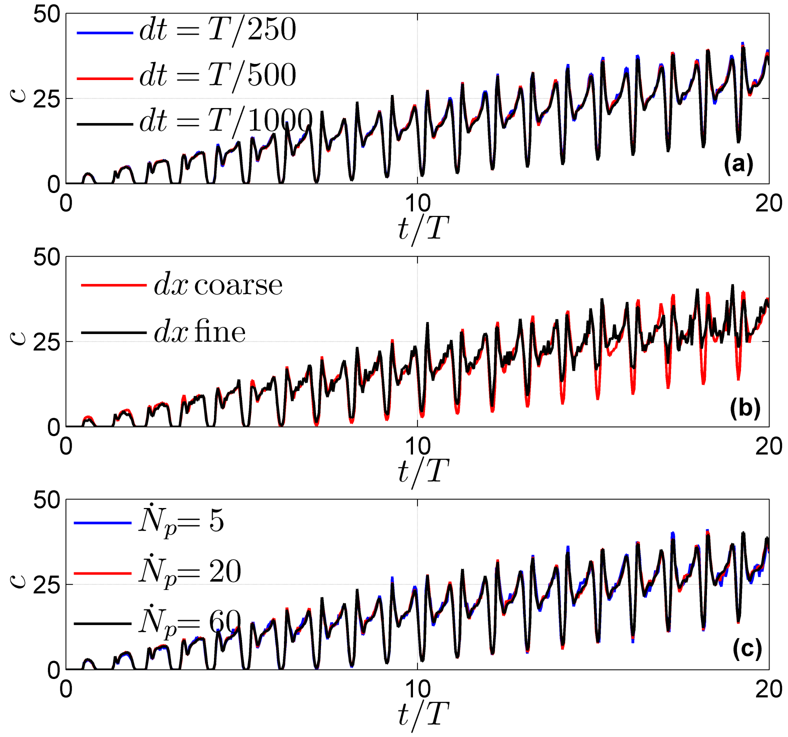

Fig 1. Drug concentrations temporal profiles obtained with different time step, grid and number of particle resolutions. Temporal profile of the normalized drug concentration obtained with perpendicular injection at P_1 and different (a) time steps; (b) mesh resolutions; (c) number of injected particles per time-step.

References

1. Haga PT. Numerical simulations of advection-dominated scalar mixing with applications to spinal CSF flow and drug transport. MSc thesis, University of Oslo. May, 2015. Available: https://www.duo.uio.no/handle/10852/45371.
