# Supplementary material for: A numerical investigation of intrathecal isobaric drug dispersion within the cervical subarachnoid space
Source: PLoS One. 2017 Mar 15;12(3):e0173680. doi: 10.1371/journal.pone.0173680 (PMC5351861; doi:10.1371/journal.pone.0173680)
Supplement: S3 Appendix — Exemplary numerical results showing drug concentration evolution after injection for test case S1. (PDF) [file pone.0173680.s003.pdf]

Supporting Information S3

A Numerical Investigation of Intrathecal Isobaric Drug Dispersion within the Cervical Subarachnoid Space

P.T. Haga[1◑], G. Pizzichelli[2,3◑], M. Mortensen[1,4¤], M. Kuchta[4], S. Heidari Pahlavian[5], E. Sinibaldi[2], B.A. Martin[6*], K.A. Mardal[1,4]

1 Center for Biomedical Computing, Simula Research Laboratory, Fornebu, Norway
2 Istituto Italiano di Tecnologia, Center for Micro-BioRobotics, Pontedera, Italy
3 Scuola Superiore Sant'Anna, The BioRobotics Institute, Pontedera, Italy
4 Dept. of Mathematics, University of Oslo, Oslo, Norway
5 Conquer Chiari Research Center, Dept. of Mech. Engineering, University of Akron, Akron, Ohio, USA
6 Dept. of Biological Engineering, The University of Idaho, 875 Perimeter Drive MS 0904, Moscow, Idaho, USA

◑These authors contributed equally to this work.
* Corresponding author: brynm@uidaho.edu

Concentration trend after injection

In the present study we considered a limited time window (20 cardiac cycles, i.e. nearly 15 s) because of the computational costs inherently associated with the adopted forward-modeling approach with physics resolved both in space and time. To qualitatively assess the effect of the injection termination, we also ran a longer simulation. In particular, we considered a 60 s time window during which we only injected over the initial 30 s. With regard to the injection parameters, we adopted those of test case S_1 (injection at P_1, θ=0°, ϕ=0°, $\bar{v}_{inj}$=6 cm/s).

Exemplary numerical results for the evolution of drug concentration are reported in Fig.1, where c represents a normalized drug concentration on a fixed cross-section at a distance of Δy from the injection point. In particular, we divided the concentration in a thin volume slice ($0.8\,mm$ thick) centered at the considered cross-section by the total average concentration in the whole domain after 10 cycles. It should be noted that c linearly increased during injection, while it tends to flatten and decrease when the injection stopped. Qualitatively speaking, these results describe the expected gradual decrease in drug concentration after injection, consistent with prior numerical results [1, 2].

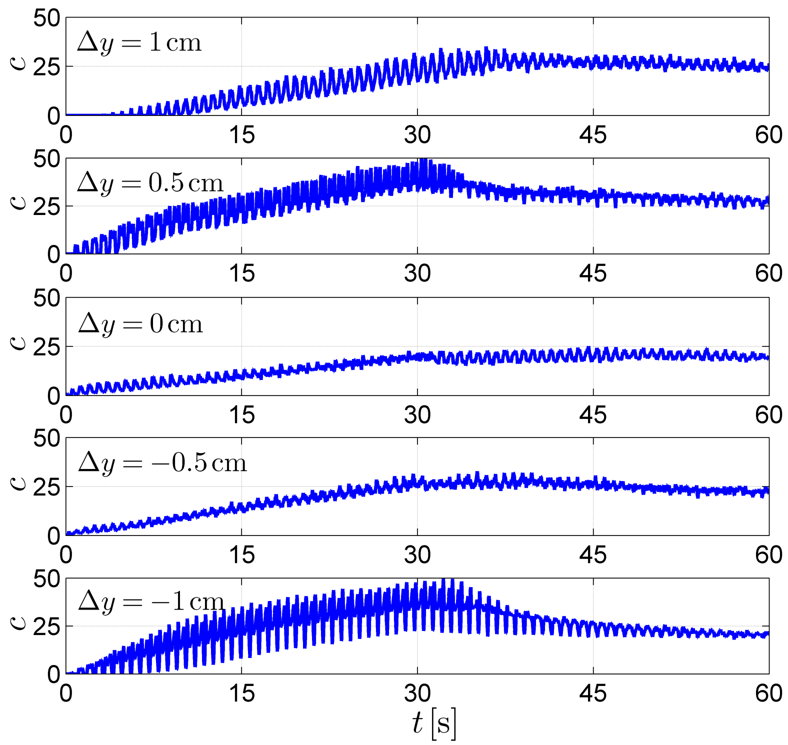

Fig 1. Particle concentration after injection. Temporal profile of the normalized drug concentration obtained with perpendicular injection at P_1 for selected distances Δy from the injection point and by stopping the injection after 30 s.

References

1. Hsu Y, Hettiarachchi HDM, Zhu DC, Linninger AA. The frequency and magnitude of cerebrospinal fluid pulsations influence intrathecal drug distribution: key factors for interpatient variability. Anesth Analg. 2012 Aug;115(2):386–394.

2. Tangen KM, Hsu Y, Zhu DC, Linninger AA. CNS wide simulation of flow resistance and drug transport due to spinal microanatomy, J Biomech. 2015 Jul;48(10):2144–2154.
